# Identification of an alpha-1 antitrypsin variant with enhanced specificity for factor XIa by phage display, bacterial expression, and combinatorial mutagenesis

**DOI:** 10.1038/s41598-021-84618-7

**Published:** 2021-03-10

**Authors:** Varsha Bhakta, Mostafa Hamada, Amy Nouanesengsy, Jessica Lapierre, Darian L. Perruzza, William P. Sheffield

**Affiliations:** 1 Centre for Innovation, Canadian Blood Services, Hamilton, ON Canada; 2 Department of Pathology and Molecular Medicine, McMaster University, HSC 4N66, 1280 Main Street West, Hamilton, ON L8S 4L8 Canada

**Keywords:** Biochemistry, Molecular biology, Molecular medicine

## Abstract

Coagulation Factor XIa (FXIa) is an emerging target for antithrombotic agent development. The M358R variant of the serpin alpha-1 antitrypsin (AAT) inhibits both FXIa and other proteases. Our aim was to enhance the specificity of AAT M358R for FXIa. We randomized two AAT M358R phage display libraries at reactive centre loop positions P13-P8 and P7-P3 and biopanned them with FXIa. A bacterial expression library randomized at P2′-P3′ was also probed. Resulting novel variants were expressed as recombinant proteins in *E. coli* and their kinetics of FXIa inhibition determined. The most potent FXIa-inhibitory motifs were: P13-P8, **H**A**STGQ**; P7-P3, **C**LE**VE**; and P2-P3′, PRS**TE** (respectively, novel residues bolded). Selectivity for FXIa over thrombin was increased up to 34-fold versus AAT M358R for these single motif variants. Combining CLEVE and PRSTE motifs in AAT-RC increased FXIa selectivity for thrombin, factors XIIa, Xa, activated protein C, and kallikrein by 279-, 143-, 63-, 58-, and 36-fold, respectively, versus AAT M358R. AAT-RC lengthened human plasma clotting times less than AAT M358R. AAT-RC rapidly and selectively inhibits FXIa and is worthy of testing in vivo. AAT specificity can be focused on one target protease by selection in phage and bacterial systems coupled with combinatorial mutagenesis.

## Introduction

Thrombosis, the blockage of intact blood vessels by occlusive clots, continues to impose a heavy clinical burden, and is responsible for one quarter of deaths world-wide^1^. Existing antithrombotic agents target either overactive platelets or coagulation factors and are all limited by their association with an increased risk of bleeding^2^. An ideal antithrombotic target would be one whose reduction in quantity and/or activity reduces thrombotic risk without elevating hemorrhagic risk. Coagulation Factor XI (FXI) has emerged as one such promising target^3^.

Patients with a genetic deficiency of FXI suffer from lower rates of ischemic stroke or deep vein thrombosis than normal controls, typically with little or no elevation in bleeding tendency^4–8^. Conversely, those with elevated concentrations of FXI in their circulation demonstrate elevated rates of venous or arterial thrombosis^9–11^. Knockout mice lacking FXI do not bleed more than normal mice upon hemorrhagic challenge and exhibit less extensive thrombi than normal mice in multiple models of arterial thrombosis^12–16^. Circulating levels of FXI can be reduced by an antisense oligonucleotide agent specific for FXI mRNA; a phase 2 trial demonstrated that this approach was superior to low molecular weight heparin in preventing thrombosis and did not increase bleeding in patients undergoing knee replacements^17^. However, it takes several weeks for the antisense approach to reduce FXI levels in the circulation.

FXI is secreted into the plasma compartment following synthesis in hepatocytes and circulates as a ~ 160 kDa dimer^18–20^. FXI is converted into enzymatically active FXIa either by activated FXII in the contact pathway or via feedback activation by thrombin; both activators cleave the R369-I370 bond^21^. FXIa is inhibited by several members of the serpin superfamily (e.g. protein Z-dependent protease inhibitor^22^, protease nexin 1^23^, alpha-2 antiplasmin^24^, and antithrombin^25^), none of which is specific for FXIa. FXIa is also inhibited by the Kunitz family inhibitor protease nexin 2, but this protein is not found in plasma at detectable levels^26^. These natural inhibitors are overwhelmed in thrombosis.

Alpha-1 antitrypsin (AAT, also known as alpha-1 proteinase inhibitor or SERPIN A1)^27–29^ reacts slowly with thrombin or FXIa, but an M358R substitution in its critical reactive centre loop (RCL) increases its rate of inhibition by over 1000-fold for both clotting factors^30,31^. We^32^ and others^33^ have shown that recombinant AAT M358R has strong antithrombotic activity in rodent thrombosis models, but it is not clear how much of this protection comes from its pro-hemorrhagic anti-thrombin or more desirable anti-FXIa activity. Additional RCL mutations can modify serpin specificity (reviewed in^34^). Recently two groups used AAT M358R as a starting point for additional modifications intended to focus the inhibitor to inhibit Activated Protein C (APC) or “thromboinflammation” mediated by kallikrein or FXIIa, inspired by structural considerations^35,36^ or lessons from small peptide studies^37^.

An alternative approach involves creating large expression libraries of AAT M358R variants and selecting candidates for reactivity with a target proteinase^38^. Previously we screened an AAT M358R library hypervariable at 5 contiguous RCL positions for reactivity with thrombin, finding two novel variants that inhibited thrombin with significantly enhanced rates^39^. In the current study we screened two phage display libraries and a bacterial expression library with FXIa to survey most residues in the AAT M358R RCL. We identified the most specific variant in each of the three probed portions of the RCL, and then combined them to find the AAT M358R variant most specific for FXIa, designated AAT-RC. This variant, which notably contained two glutamic acid substitutions proximal and distal to M358R, was 279-fold more selective for FXIa than thrombin, relative to AAT M358R, and was less selective for FXIIa, FXa, APC and kallikrein by factors ranging from 36- to 143-fold.

## Results

### General approach

**Figure 1:**
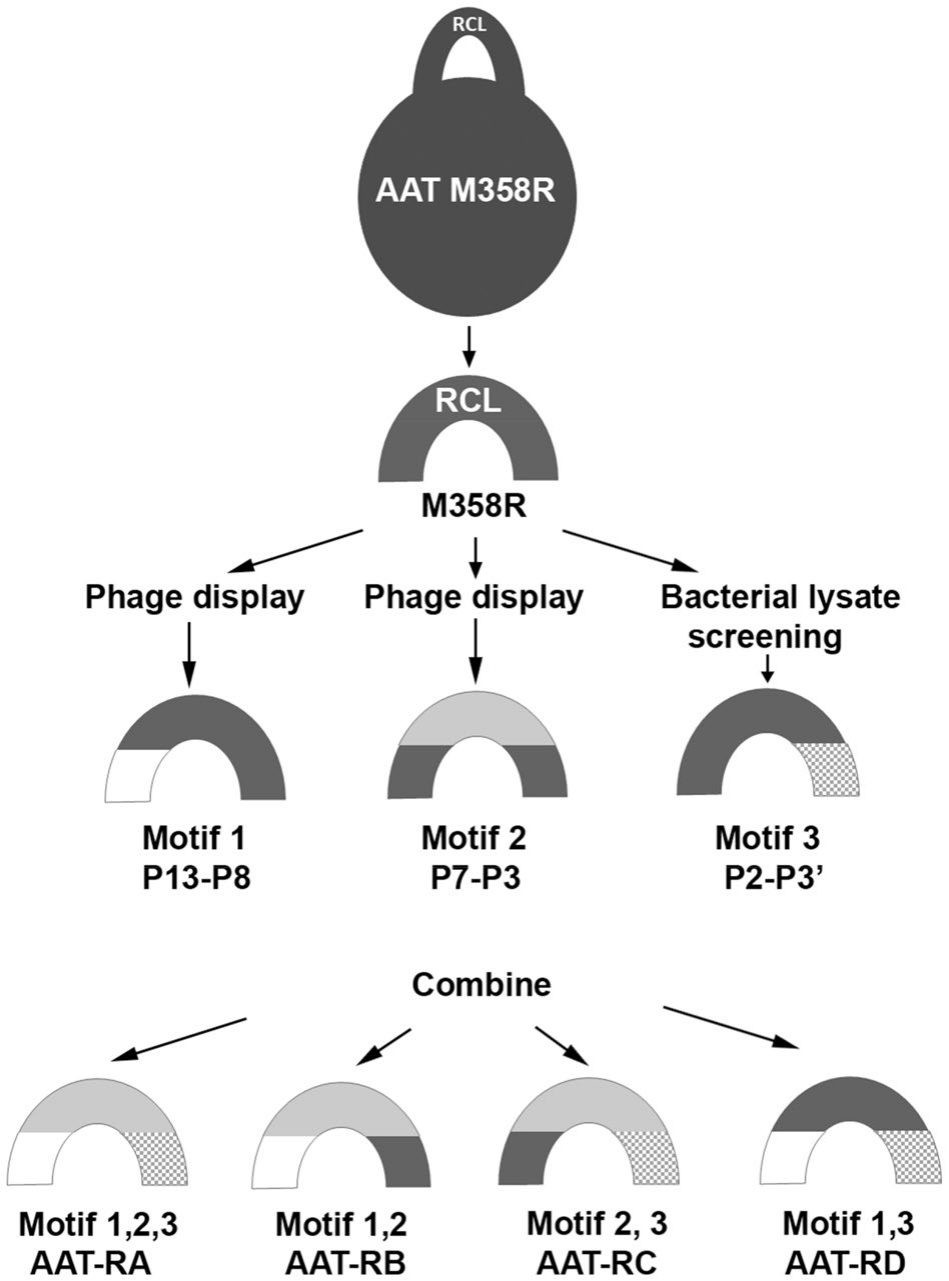
Schematic diagram of screening and mutagenesis approaches used in this study. “Kettle ball” diagram of AAT M358R shows the body of the serpin (circle) with a protruding reactive centre loop (RCL, black). P13-P8 (with P12 fixed as A353) residues were probed by biopanning an AAT phage display library randomized at these positions with FXIa to select the most active Motif 1 (white). Similarly, P7-P3 residues were independently probed in the same way to select the most active Motif 2 (grey). P2-P3′ residues were fixed at P2 to P1′ as PRS (P357/M358R/S359) and P2′-P3′ residues were probed by testing lysates of a hypervariable AAT bacterial expression library for high FXIa binding and low thrombin binding to select the most FXIa-specific Motif 3 (hatched). Motifs 1, 2, and 3 (AAT-RA), 1 and 2 (AAT-RB), 2 and 3 (AAT-RC), and 1 and 3 (AAT-RD) were then assembled for bacterial expression as hexahistidine-tagged AAT M358R variants, purified, and characterized kinetically.

We conducted three screening exercises, seeking AAT RCL sequences found in AAT variants with the greatest activity against, and specificity for, FXIa. As shown in Fig. 1, this exercise spanned the RCL from E346-P361 (P13-P3′ in the nomenclature of Schechter and Berger, where the reactive centre bond R358-S359 is P1-P1′)^40^. No more than 5 RCL codons can be randomized at a time to generate libraries that can be productively probed by phage display^39^, and no more than two codons at a time in more labour-intensive bacterial lysate screening protocols^41^. We therefore probed the RCL in segments of five contiguous codons: P13-P8; P7-P3; and P2-P3′. We allowed any nucleotide at all three positions in all codons that were randomized to generate libraries (see Table S1). We first probed P7-P3 residues, in a previously constructed library^39^.

### P7-P3 phage display library screening

**Figure 2:**
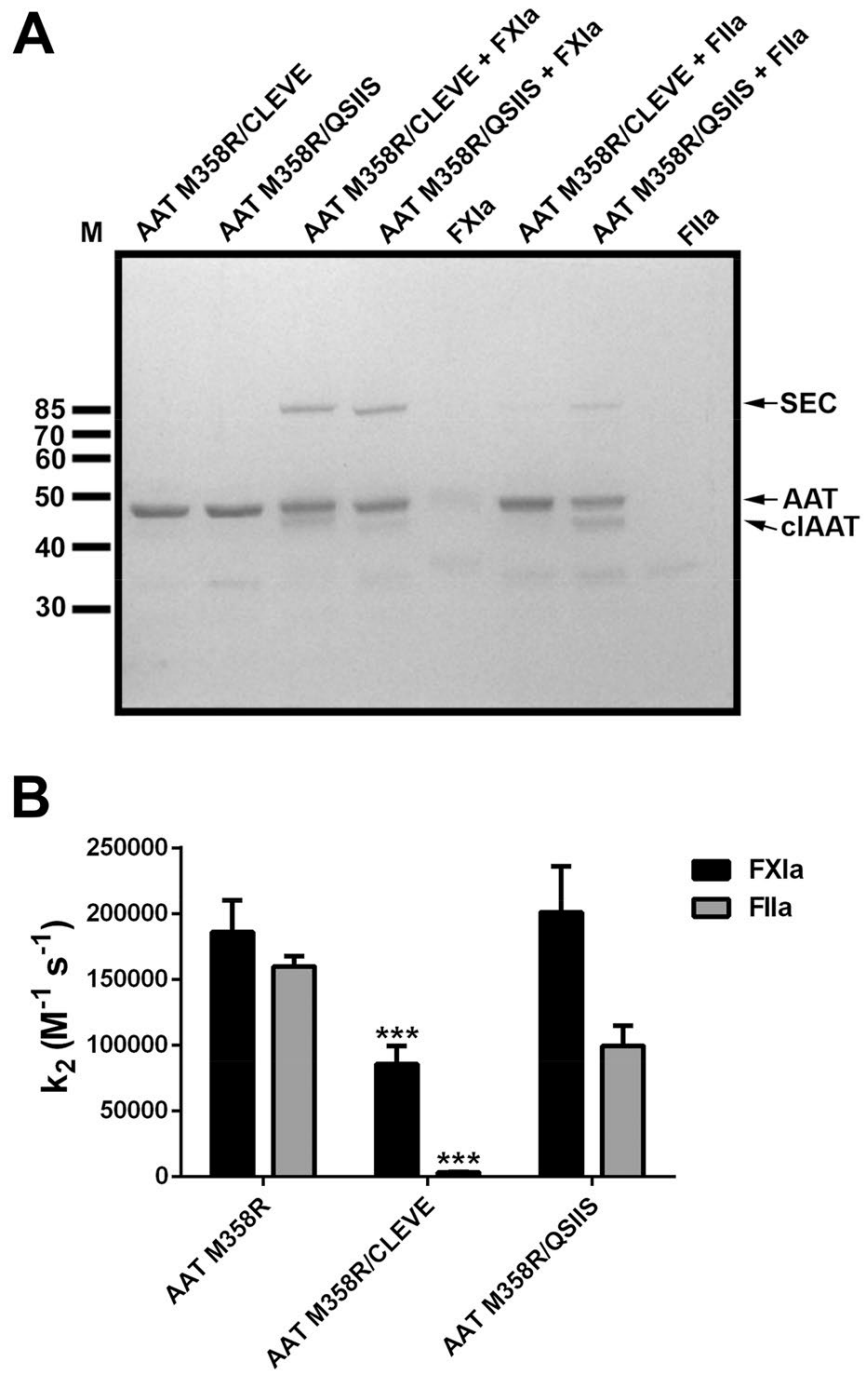
Reactions of AAT P7-P3 motif 2 variants with FXIa and thrombin. Panel (**A**): Electrophoretic assay of reactions of purified AAT M358R/CLEVE or AAT M358R/QSIIS with FXIa or thrombin (FIIa). AAT variants (1.0 µM) were reacted with protease (0.1 µM) for 2 min at 37 °C. Arrows (right) highlight the positions of serpin-enzyme complexes (SEC), uncleaved AAT (AAT), and AAT cleaved in its reactive centre loop (clAAT) on a stained reduced SDS–polyacrylamide gel. M, molecular weight markers are shown at left in kDa. Panel (**B**): Second order rate constants of inhibition (k_2_) of AAT variants with FXIa (black) and FIIa (grey) are shown as the mean of 3–7 determinations ± SD.

After five rounds of biopanning with FXIa, we determined the RCL sequence of 41 plaque-purified phages. The identity and frequency of the selected P7-P3 codons were: 13 QSIIS; 7 CLEVE; 3 FAASE; 2 PIVAE; 2 YLDSL; and 14 different single pentapeptide sequences. As the most abundant selected sequences, QSIIS and CLEVE were selected for further characterization in soluble form, using an arabinose-inducible bacterial expression system. As shown in Fig. 2A, both AAT M358R/CLEVE and AAT M358R/QSIIS variants formed SDS-stable serpin-enzyme complexes (SEC) with FXIa more readily than with thrombin, in single time point reactions visualized on gels. Kinetic analysis (Fig. 2B) revealed that the k_2_ for FXIa inhibition of AAT M358R/CLEVE was reduced 2.1-fold versus AAT M358R (from 1.9 ± 0.2 × 10^5^ M^-1^ s^-1^ to 9 ± 1 × 10^4^ M^-1^ s^-1^) but that of AAT M358R/QSIIS was unchanged. In contrast, the rate of thrombin inhibition was reduced 48-fold for AAT M358R/CLEVE (from 1.60 ± 0.08 × 10^5^ M^-1^ s^-1^ to 3.3 ± 0.1 × 10^3^ M^-1^ s^-1^) but less than twofold for AAT M358R/QSIIS (to 1.0 ± 0.2 × 10^5^ M^-1^ s^-1^). The ratio of the rate constants (i.e. the selectivity) for FXIa over thrombin of AAT M358R/CLEVE was therefore increased 23-fold versus AAT M358R, compared to only 1.6-fold for AAT M358R/QSIIS. On this basis CLEVE was chosen as the optimal P7-P3 sequence (Motif 2) arising from phage display.

### P13-P8 phage display library screening

Having demonstrated that phage display and FXIa-mediated biopanning could be productively employed to find AAT M358R variants inhibiting FXIa with enhanced selectivity using the P7-P3 library, we applied the same approach to the proximal end of the RCL, from P13-P8. As before, we randomized 5 codons, leaving P12 fixed as A347, due to the strong conservation of Ala at P12 among inhibitory serpins^42,43^. Five rounds of biopanning with or without FXIa were carried out, and the selected populations were characterized by deep sequencing. We previously showed that the lists of the most abundant sequences identified via sequencing of selected clones (as was done for the P7-P3 library above) or deep sequencing (employed for the P13-P8 library) exhibited considerable overlap, in that case for the AAT P7-P3 library probed with thrombin^39^. As shown in Table S2, two sequences accounted for 90.3% of the > 24,000 reads obtained from the FXIa-selected library, encoding HASTGQ (57%) and IAKYAT (33.3%). The third most-abundant sequence (FASSSG), unlike HASTGQ and IAKYAT, was also found at high abundance in the mock-selected library and was not further investigated. It is possible that we might have identified HASTGQ earlier in the selection process had we employed deep sequencing on the phage populations from earlier rounds, or even the first round alone.

HASTGQ and IAKYAT were characterized by expression in a bacterial expression system, like P7-P3 variants QSIIS and CLEVE, but differing slightly from the arabinose-inducible system previously employed in that a cleavable glutathione-sulfotransferase (GST) tag was exploited for improved yield. Both systems produced N-terminally hexahistidine-tagged proteins. The protein profile of AAT M358R from this system at intermediate and final steps is shown in Fig. S1. The k_2_ values for FXIa inhibition for AAT M358R produced in the arabinose-inducible system (1.8 ± 0.4 × 10^5^ M^-1^ s^-1^) and the cleavable GST fusion system (1.7 ± 0.3 × 10^5^ M^-1^ s^-1^) did not differ significantly.

As shown in Fig. S2, AAT M358R/HASTGQ and AAT M358R/IAKYAT both formed SDS-stable inhibitory complexes with FXIa more readily than they formed the corresponding complexes with thrombin. Because HASTGQ was expressed at higher levels than IAKYAT, the former sequence was chosen as the optimal P13-P8 sequence (Motif 1) arising from phage display.

### P2-P3′ bacterial expression library screening

For the final, distal portion of our RCL survey, we elected to fix P2/P1/P1′ as PRS. Two groups previously reported reduced expression in bacteria for different P2 variants in the M358R context^44,45^. P1 at M358R was fixed because of the large increment in FXIa inhibition accompanying this key substitution^31^. P1′ was fixed as S359 because two reports had indicated increased anti-APC and/or increased anti-thrombin activity for variants in the M358R/S359 context^45,46^. These considerations reduced the residues under investigation to P2′ and P3′; randomizing only two positions meant that direct expression of the hypervariable library in bacteria and screening of bacterial lysates^41^ was feasible, unlike in the more extensive screens we undertook covering P13-P3. Three hundred colonies were screened in pools of ten, and “hits” corresponding to high FXIa binding and low thrombin binding in microtiter plate assays were deconvoluted and repeated at the single colony level. Two variants, AAT M358R/PRSTE and AAT M358R/PRSLD, were selected for further characterization. Fig. S3A shows that both variants readily formed complexes with FXIa and not thrombin, but kinetic experiments (Fig. S3B) showed that the k_2_ for FXIa inhibition was significantly greater for AAT M358R/PRSTE than AAT M358R/PRSLD (4.5 ± 0.5 × 10^4^ vs. 3.8 ± 0.3 × 10^4^, *p* < 0.001). On this basis PRSTE was selected as the optimal distal RCL sequence arising from our P2-P3′, or more specifically P2′/P3′, library screening scan (Motif 3).

### Comparison of single motif variants

**Figure 3:**
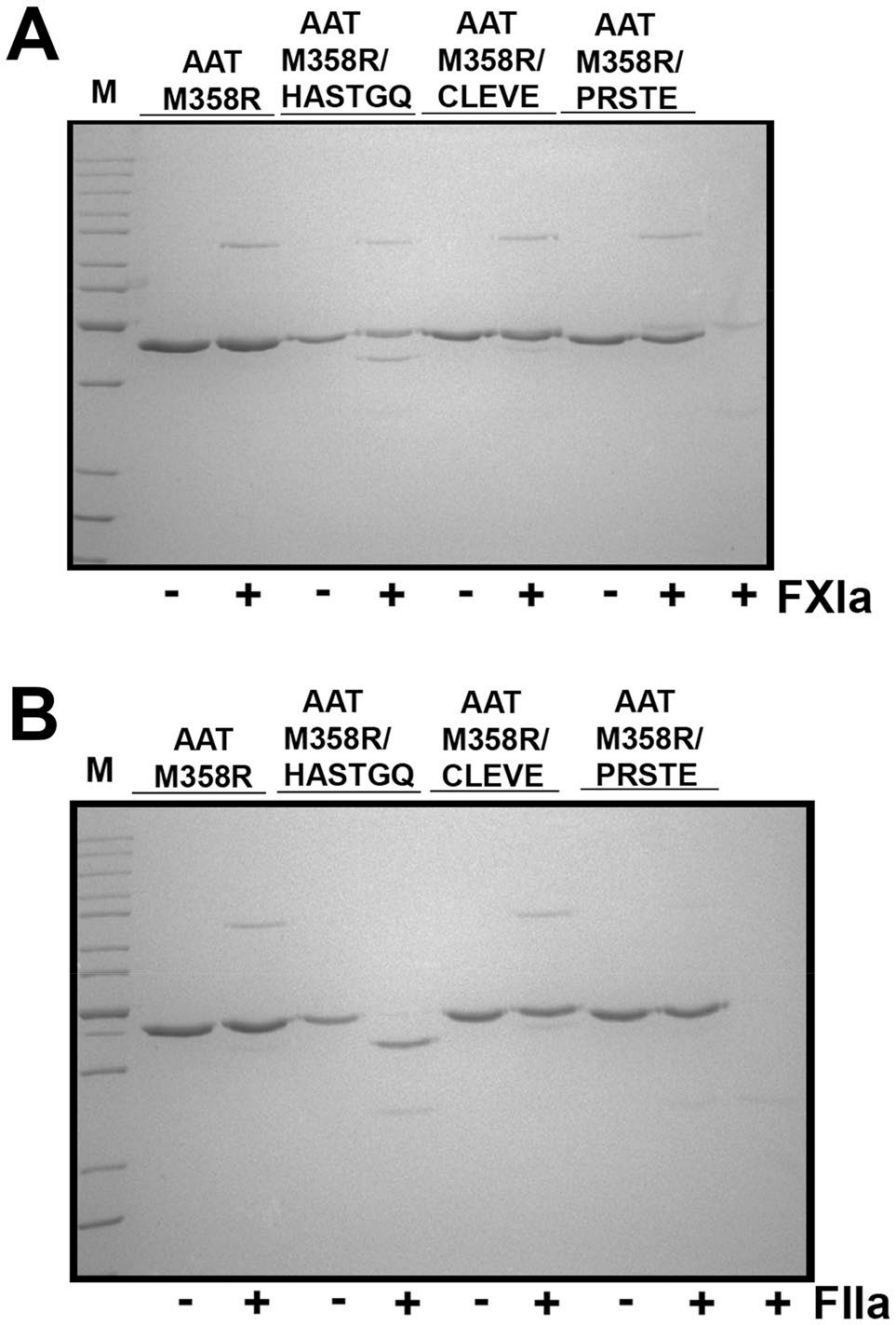
Reactions of AAT M358R and variants with RCL substitutions comprising motifs 1–3 with FXIa and thrombin. AAT variants (at 1.0 µM, identified above the lanes) were incubated at 37 °C for one minute with ( +) or without ( −) 0.1 µM FXIa (Panel **A**) or five minutes with ( +) or without ( −) 0.1 µM FIIa (Panel **B**) prior to SDS-PAGE under the same conditions and with the same markers as in Fig. 2.

Prior to combining the single motif variants, they were contemporaneously expressed and purified in the GST system, with AAT M358R. As expected, and as shown in Fig. 3, reactivity with FXIa was maintained to a greater extent than with thrombin, with some increases in substrate behavior. Quantitatively, as shown in Table 1, AAT M358R/CLEVE and AAT M358R/PRSTE showed similar 25- and 34-fold increases in selectivity for FXIa over thrombin; selectivity could not be calculated for AAT M358R/HASTGQ because inhibition of thrombin by that variant was not detected, even when employing 100- to 200-fold molar excesses of variant serpin over protease.

**Table 1:** Kinetic parameters for single and double motif AAT variants compared to AAT M358R.

Protein	k_2_ for FXIa (X 10^5^ M^-1^ s^-1^)	Stoichiometry of Inhibition (SI)	k_2_ for Thrombin (X 10^5^ M^-1^ s^-1^)	Selectivity (k_2_ for FXIa/ k_2_ for Thrombin)	Fold increase in thrombin selectivity vs AAT M358R
AAT M358R	1.6 ± 0.2^c^	2.1 ± 0.3^a^	2.1 ± 0.6^c^	0.76	–
AAT M358R/HASTGQ	0.34 ± 0.04^a^	12.7 ± 0.9^a^	ND*	ND*	ND*
AAT M358R/CLEVE	1.5 ± 0.1^a^	6.6 ± 0.2^a^	0.081 ± 0.006^a^	19	25
AAT M358R/PRSTE	0.41 ± 0.07^a^	3.3 ± 0.1^a^	0.012 ± 0.001^a^	26	34
AAT-RC	0.7 ± 0.1^c^	7 ± 1^c^	0.0033 ± 0.0009^b^	212	279
AAT-RD	0.20 ± 0.07^a^	7.0 ± 0.4^a^	ND*	ND*	ND*

The mean of 5^a^, 7^b^, 9^c^, or 10^d^ determinations ± one standard deviation is shown, for kinetic parameters for single and double motif AAT variants, compared to AAT M358R.

### Combining motifs

**Figure 4:**
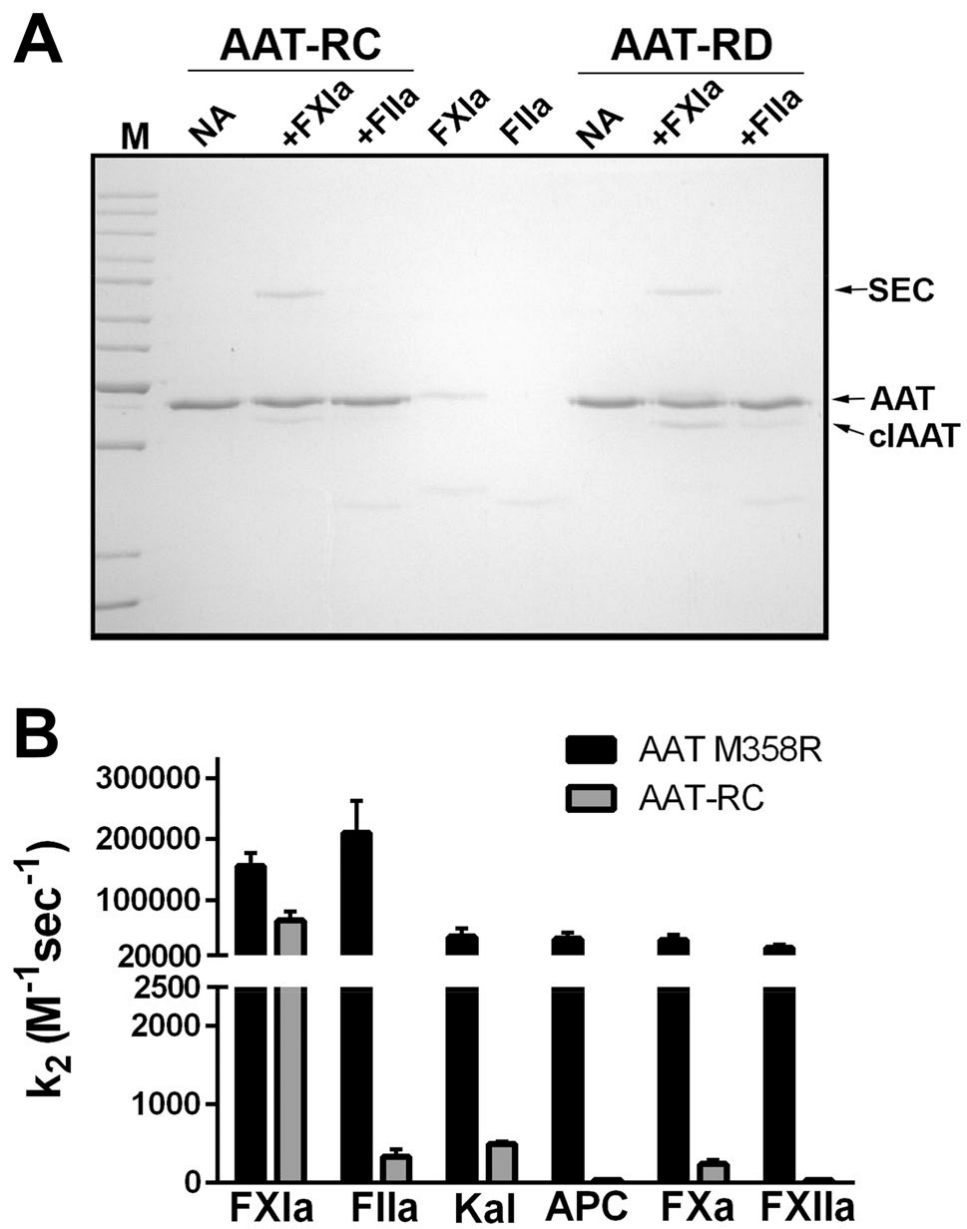
Reactions of AAT M358R, AAT-RC, and AAT-RD variants with FXIa and FIIa. Panel A, AAT-RC or AAT-RD variants (at 1.0 µM, identified above the lanes) were incubated at 37 °C for one minute with (+ FXIa or + FIIa) or without (no addition, NA) 0.1 µM FXIa or for five minutes with or without 0.1 µM FIIa (Panel **A**), prior to SDS-PAGE under the same conditions and with the same markers as in Figs. 2 and 3. Panel (**B**) shows second order rate constants of inhibition (k_2_) of AAT M358R (black) or AAT-RC (grey) with different proteases identified on the x axis (F(actor) XIa, FIIa, FXa, FXIIa, kallikrein (Kal), or Activated Protein C (APC)). Bars are the mean of 6–9 determinations ± SD. Note the break on the y axis.

As shown in Fig. 1 and Table 2, the three motifs identified in the screening exercises were next all combined (AAT-RA) or combined two at a time, as follows: AAT-RB, motifs 1 and 2; AAT-RC, motifs 2 and 3; or AAT-RD, motifs 1 and 3. To gain a preliminary understanding of the effects of combined motif expression, the four constructs were expressed using the arabinose inducible system. AAT concentrations were normalized by immunoassay, and variants were reacted with FXIa (Fig. S4A) or thrombin (Fig. S4B). While no variant reacted detectably with thrombin, some complex formation was detectable with FXIa in all cases, although AAT-RC and AAT-RD were clearly more effective FXIa inhibitors than AAT-RA or AAT-RB. Accordingly, these two variants were expressed in the GST expression system and purified. Figure 4A shows a gel of the reactions of AAT-RC and AAT-RD with FXIa and thrombin, in which the similar reactivity of these variants with FXIa (and not thrombin) seen in the preliminary immunoblot experiments was reproduced.

**Table 2:** RCL Sequences of AAT M358R and variants examined in this study.

Protein name	RCL sequence (P13-P3′)
AAT M358R	**EAAGAMFLEAIPRSIP**
AAT M358R/HASTGQ	H**A**STGQ**FLEAIPRSIP**
AAT M358R/CLEVE	**EAAGAM**C**LE**VE**PRSIP**
AAT M358R/PRSTE	**EAAGAMFLEAIPRS**TE
AAT-RA	H**A**STGQC**LE**VE**PRS**TE
AAT-RB	H**A**STGQC**LE**VE**PRSIP**
AAT-RC	**EAAGAM**C**LE**VE**PRS**TE
AAT-RD	H**A**STGQ**FLEAIPRS**TE

Primary amino acid sequences of various AAT M358R derivatives between P13 (E346) and P3′ (P361) are shown. Bold indicates AAT M358R sequence; regular indicates mutated residues.

### Kinetic analysis versus FXIa and thrombin

All single motif AAT M358R variants and double motif variants AAT-RC and AAT-RD were purified to homogeneity using the cleavable GST expression system and characterized kinetically. As shown in Table 3 and Fig. 4B, mean k_2_ values for FXIa inhibition among these novel variants ranged from 2.0 × 10^4^ M^-1^ s^-1^ (AAT-RD) to 1.5 × 10^5^ M^-1^ s^-1^ (AAT M358R/CLEVE). Mean stoichiometry of inhibition (SI) values for FXIa inhibition ranged from 3.3 (AAT M358R/PRSTE) to 12.7 (AAT M358R/HASTGQ). Mean k_2_ values for thrombin inhibition, where they could be calculated, ranged from 8.1 × 10^3^ M^-1^ s^-1^ (AAT M358R/CLEVE) to 3.3 × 10^1^ M^-1^ s^-1^ (AAT-RC). The variant with the greatest selectivity for FXIa over thrombin was therefore AAT-RC, with a selectivity of 212, a 279-fold increase in selectivity for FXIa over thrombin relative to AAT M358R. This enhanced selectivity came at a cost of a 2.3-fold decrease in the rate of FXIa inhibition and a 3.3-fold increase in the SI. Given the slower rate of FXIa inhibition of AAT-RD and our inability to measure a rate of thrombin inhibition, we selected AAT-RC over AAT-RD for further characterization.

**Table 3:** Kinetic parameters for AAT-RC and AAT M358R with various proteases.

Protease	k_2_ AAT M358R (X 10^5^ M^-1^ s^-1^)	k_2_ AAT-RC (X 10^5^ M^-1^ s^-1^)	Selectivity of AAT M358R (k_2_ for FXIa/k_2_ for protease)	Selectivity of AAT-RC (k_2_ for FXIa/k_2_ for protease)	Fold difference in selectivity (AAT-RC/ AAT M358R)
FXIa	1.6 ± 0.2^d^	0.7 ± 0.1^d^	–	–	–
Thrombin	2.1 ± 0.6^c^	0.0033 ± 0.0009^b^	0.76	212	279
Kallikrein	0.4 ± 0.1^d^	0.0049 ± 0.0004^b^	4.0	143	36
APC	0.4 ± 0.1^c^	0.003 ± 0.002^a^	4.0	233	58
FXa	0.35 ± 0.08^d^	0.0024 ± 0.0005^d^	4.6	292	63
FXIIa	0.23 ± 0.04^b^	0.0003 ± 0.0002^d^	7.0	1000	143

The mean of 4^a^, 7^b^, 8^c^, or 9^d^ determinations ± one standard deviation is shown, for kinetic parameters for AAT-RC and AAT M358R with various proteases.

### Kinetics of serine protease inhibition by AAT-RC versus AAT M358R

Having demonstrated that AAT-RC was much more reactive with FXIa than thrombin, we sought to determine if this enhanced specificity also extended to other serine proteases involved in thrombosis and/or inflammation. As shown in Table 3, AAT-RC was on average 212-fold more selective for FXIa than thrombin, a difference of 279-fold versus AAT M358R. It also demonstrated enhanced selectivity for FXIa over four other serine proteases compared to AAT M358R, ranging from being 1000-fold more selective for FXIa than FXIIa to 143-fold more selective for FXIa than kallikrein.

### Effects of AAT-RC or AAT M358R on clotting assays

**Figure 5:**
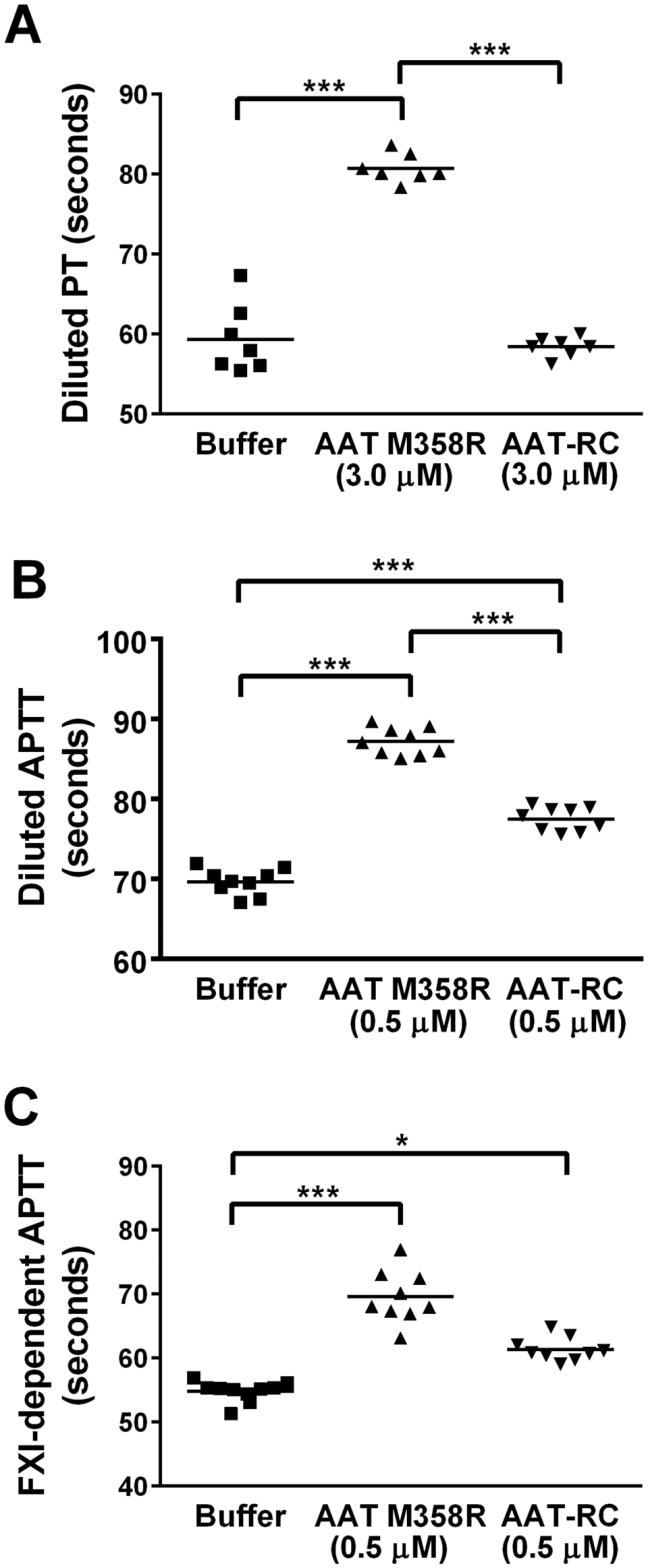
Effects of AAT M358R or AAT-RC on modified hemostasis screening tests. Buffer or purified AAT M358R was introduced at either 0.5 µM or 3.0 µM final concentrations (as labelled on x axes) into diluted PT (Panel **A**), diluted APTT (Panel **B**), or FXI-dependent APTT assays. Note y axis does not commence at 0 s. Each point is a single determination. Horizontal capped lines linking groups indicate statistical differences by ANOVA with post-tests: *p < 0.05; **p < 0.01; and ***p < 0.001.

To extend our characterization of AAT-RC beyond purified systems and into plasma, we next investigated the effects of AAT variant supplementation on plasma clotting times. We elected to use variations on the Prothrombin Time (PT) and the Activated Partial Thromboplastin Time (APTT) assays, in which activation of clotting was slowed by dilution of the PT reagent (containing tissue factor for extrinsic pathway activation) or the APTT reagent (containing silicates for intrinsic pathway activation)^47^. Slowing activation facilitates the measurement of inhibition^37^. As shown in Fig. 5A, supplementing plasma with AAT M358R (to 3.0 µM final concentration) significantly elevated the diluted PT 1.4-fold, from 58 ± 2 s to 81 ± 2 s (*p* < 0.001); in contrast, AAT-RC had no effect, with an observed PT of 58 ± 1 s. Figure 5A and 5B show that AAT-RC supplementation prolonged two different variations of the APTT assay, but in both cases to a lesser extent than AAT M358R. Supplementation of the diluted APTT to 0.5 µM AAT-RC elevated clotting times to values intermediate between buffer (70 ± 2 s) and 0.5 µM AAT M358R (87 ± 2 s) of 78 ± 1 s (Fig. 5B). The same comparative ranking was observed in a variation of the APTT in which clotting was rendered FXIa-dependent using FXI-deficient plasma supplemented with FXIa (Fig. 5C). These results suggest that AAT-RC has a less generalized anticoagulant effect in human plasma than AAT M358R and suggest that its in vivo use could be associated with less generalized bleeding risk.

## Discussion

This study was prompted by the unmet clinical needs for FXIa inhibition and by our previous demonstrations that: novel variants of AAT M358R could be selected using phage display^39^ or lysate screening^41^; and that AAT M358R-expressing T7 phages could be selected by FXIa-mediated biopanning from a population of phages not expressing this serpin^38^. We hypothesized that using FXIa as a bait protein in an iterative strategy would provide enough selective pressure to find AAT M358R variants with novel RCL substitutions that would be more selective for FXIa inhibition than the starting parental molecule. We took a sectoral approach because it is not technically feasible to construct and probe expression libraries containing a fully randomized RCL^39^. Although the catalytic domain of FXIa (FXIac) has been co-crystallized with small protein inhibitors like the Kunitz Protease Inhibitor (KPI) domain of protease nexin 2^48^ or ecotin^49^, no crystal structure of either an encounter complex or a serpin-enzyme complex of FXIac with AAT M358R was available to support a structural design strategy.

The most specific anti-FXIa inhibitor in the first sector we probed, P7-P3, was AAT M358R/CLEVE, which featured substitutions at 3 of 5 positions: F352C; A355V; and I356E. Selection of this variant by FXIa during biopanning supported our hypothesis, in that this variant was 25-fold more selective for FXIa than for thrombin, compared to AAT M358R; the k_2_ for FXIa was almost unchanged (reduced 6.25%), while the SI was elevated 3.3-fold, indicating that the cost of the increased specificity was an increase in substrate-like behaviour. Few investigators have sought to enhance the specificity of AAT M358R for FXIa; the only previously described partially overlapping variant was a P4-P2 IEG tripeptide substitution in AAT M358R, which included the I356E substitution we selected in AAT M358R/CLEVE (see Table S3 for alignments of the RCL sequences of all AAT variants discussed herein, plus antithrombin^50^ and protease nexin 1^51^). Izaguirre et al*.* found that this substitution, recreating a FXa cleavage site in prothrombin, increased the rate of FXa inhibition by the variant twofold relative to AAT M358R, but decreased the rate of thrombin inhibition by 1200-fold; reactivity with FXIa was not assessed^52^. A variant with an SMT substitution between P4-P2 that did not overlap with CLEVE was reported by de Maat et al*.* as increasing the rate of FXIa or APC inhibition 1.8-fold, increasing that of kallikrein 8.9-fold, and not changing that of FXIIa^37^.

Phage display of the proximal hinge of the AAT M358R RCL (P13-P8) yielded variant AAT M358R/HASTGQ, in which all 5 of the variant residues had been altered. Most substitutions were relatively conservative, such as the P11-P9 swap of STG for AGA, involving smaller, albeit more polar amino acid substitutions. At P8, glutamine and methionine are both uncharged amino acids with similar volumes. The largest change was found at P13 (E346H). A similarly non-conservative substitution at the adjacent P14 residue (T345R) converted AAT M358R into a substrate of thrombin^53^; the same alteration in wild-type AAT also increased substrate behaviour versus pancreatic elastase^54^. AAT M358R/HASTGQ exhibited the greatest SI of the variants reported in this study. These observations are consistent with the role of this portion of the RCL as a hinge which must insert into the underlying β-sheet A when AAT translocates attacking proteases to the opposite pole of the molecule in the serpin inhibitory mechanism; the speed of insertion correlates with the outcome of the reaction, yielding either cleaved serpin and free protease (substrate pathway) or cleaved serpin and trapped protease (inhibitor pathway)^54–57^. It is not readily apparent why this variant would have been selected by FXIa over the parental AAT M358R, as we observed; however it is possible that the HASTGQ substitutions indirectly stabilized the AAT M358R/HASTGQ RCL and provided a more optimal FXIa encounter complex in the phage-displayed serpin context. The variant was certainly more specific for FXIa than thrombin, given that we could not detect a measurable rate of thrombin inhibition by AAT M358R/HASTGQ.

For the final sector of the RCL that we probed, P2-P3′, we elected to focus only on P2′-P3′. We used a lysate screening method to search for variants with strong binding of FXIa and weak binding of thrombin; unlike in the iterative phage display screens, no selective pressure was applied to reduce the diversity of the variant pool in rounds. The IP to TE substitution that we selected has not been previously examined, although Hopkins et al*.*^45,46^ reported that the corresponding LN substitution in AAT M358R enhanced the specificity of this variant for thrombin over APC, and we reported similar findings for a TQ substitution^58^. Given that both this variant and AAT M358R/PRSTE shared the same residue at P2′, this suggests that the enhanced selectivity of our third motif is due primarily to the P361E substitution at P3’.

We next asked whether combining the independently selected motifs would yield increases in selectivity for FXIa inhibition. Qualitatively, combining all three motifs (AAT-RA) or the HASTGQ and CLEVE motifs (AAT-RB) led to sub-optimal inhibition of FXIa. While we were unable to measure the rate of thrombin inhibition of AAT-RD (HASTGQ combined with PRSTE), AAT-RC showed the greatest retention of anti-FXIa activity and the lowest residual anti-thrombin activity. Combining the motifs in the AAT-RC context led to much greater increase in selectivity for FXIa (279-fold versus AAT M358R) than the sum of the increases separately seen for AAT M358R/CLEVE and AAT M358R/PRSTE (25- and 34-fold respectively, or 59-fold combined). Similarly, combining the HASTGQ motif with PRSTE in AAT-RD reduced the SI from ~ 12 to ~ 7; this indicates positive cooperativity rather than dominance by the substrate-promoting first motif. Cooperativity has been previously noted by Hopkins et al. in an extensive mutagenic study in which the RCL of antithrombin was substituted for that of AAT M358R, and then back-mutated to generate a family of less modified AAT constructs; changes in different parts of the RCL had greater effects when combined than when separate, especially on APC reactivity^46^. We also observed possible negative cooperativity between motifs 1 and 2, as evidenced by the qualitatively lower anti-FXIa activity of AAT-RA and AAT-RB, where these motifs were combined, than in AAT-RC or AAT-RD, although we did not characterize AAT-RA and AAT-RB reactions quantitatively.

The observed cooperativity also vindicates the screening strategy we employed, as opposed to an alternative strategy in which RCL residues would have been randomized one at a time, and the most FXIa-reactive variant selected at each position independently. Another alternative, that of fixing a FXIa-reactive variant at whatever amino acid was optimal at one end of the RCL, and then processing to the other end, would have a better chance of capturing productive cooperative interactions, but would also be highly labour- and time-intensive. In contrast, our screening, selection and combination strategy yielded a highly FXIa-specific inhibitor. Although we cannot claim to have exhaustively probed the sequence space, given that our libraries were not diverse enough to permit the high multiplicity of testing that would give confidence that all possibilities in the libraries had been sampled^39^, the approach did yield AAT-RC, an AAT M358R variant more selective than the parental molecule for FXIa over not only thrombin but also all four other serine proteases tested. Notably, this goal was achieved without the use of negative selection in our biopanning efforts.

The enhanced selectivity for FXIa of AAT-RC was achieved at the relatively low cost of a 2.3-fold decrease in the rate of FXIa inhibition and a 3.3-fold increase in the SI relative to AAT M358R. Polderdijk et al. reported a sevenfold decrease in the rate of APC inhibition for KRK AAT, an AAT M358R variant in which anti-APC activity was largely preserved, thrombin inhibition was abolished, and the rate of FXIa and FXa inhibition was reduced 850-fold and 360-fold, respectively^35^. SI values were not reported; gel-based analyses suggest that there was detectable, but minimal substrate behaviour with the intended target protease, APC. De Maat et al*.* made a slight modification to the previously mentioned SMTRS AAT M358R variant, to SMTRV, spanning two of the three motifs explored in this study. This variant was 2.5-fold more rapid a kallikrein inhibitor than AAT M358R, and a 20-fold less rapid plasmin inhibitor; these targeted enhancements came at a cost of a 1.8-fold reduction in the rate of inhibition of FXIa and a 1.3-fold reduction in FXIIa inhibition, as part of an ambitious effort to make AAT M358R a specific inhibitor of contact pathway proteases^37^. SI was not determined. Both KRK AAT and SMTRV were demonstrated to be effective in vivo in reducing bleeding in FIX knockout mice^35^ and limiting thrombosis and inflammation in normal mice, respectively^37^.

AAT M358R inhibits both PT and APTT human plasma clotting tests. Using the same diluted variations of these tests as de Maat et al*.*, we found that AAT-RC had no effect on the PT, like variant SMTRV; similarly it had an intermediate effect in the APTT, prolonging clotting time to a lesser extent than AAT M358R, again replicating the previously reported pattern with SMTRV^37^. That the prolongation of the dilute APTT by AAT-RC likely arose due to its anti-FXIa activity was supported by our finding that AAT-RC prolonged the APTT when it was rendered FXI-dependent via the use of FXI-depleted plasma. AAT-RC therefore resembles a variant AAT M358R that was antithrombotic in vivo in its clotting profile in human plasma, rendering it worthy of in vivo investigation in animal models. One question to be answered prior to in vivo experimentation, however, is whether all five substitutions in AAT-RC are necessary for the enhanced specificity for FXIa; it is probable that the full effect could be obtained with an AAT-RC derivative with only I356E/P361E substitutions.

The I356E/P361E substitutions are the most dramatic alterations correlating with the enhancements in FXIa specificity achieved in this study. A FXIa preference for glutamic acid at P3 has been previously noted in experiments in which an EPR tripeptide sequence was positioned N-terminal to the thrombin inhibitor hirudin, to provide an activation site^38,59^; this sequence has also been reported to be cleaved by FXIa in an amyloid beta protein precursor^60^ and is employed, in chloromethyl ketone form, as part of a chromogenic substrate with specificity for FXIa^61^.

The two glutamic acid substitutions at P3 and P3′ uncovered by our strategy were not suggested by the FXIac-KPI crystal structure^48^. KPI features two loops interacting with FXIac, one of which, T11-R20 (TGPCRAMISR), forms intimate contacts with the FXIac active site; R15 interacts with D189 of the FXIac catalytic triad and is therefore designated P1. A P13E variant at P3 bound FXIa ninefold less avidly than wild-type KPI. At P3′, a hydrophobic pocket has been suggested to be important, formed by M17 (P2′), I18 (P3′) and F34, which would not be expected to accommodate I18E^48^. This loop in KPI is therefore only partially overlapping with the AAT M358R or AAT-RC RCL in the serpin context.

Early interest in developing AAT M358R as a therapeutic agent was set back by unfavourable results in primate models^62^ and the realization that this inhibitor was more promiscuous with respect to protease specificity than originally thought^63^. Additional substitutions in the RCL have been employed to generate AAT M358R variants under investigation as treatments for hemophilia^35^ and inflammatory disorders^37^. AAT-RC could join these modern engineered AAT variants in pre-clinical development after assessment in animal models of thrombosis and hemorrhage. Our use of phage display to identify AAT-RC is only the second reported use of this technology to change the properties of AAT M358R^39^, and the second serpin to be investigated by phage display, after Plasminogen Activator Inhibitor 1 (PAI-1)^64–68^. Thus, our approach may have general utility in protein engineering of other serpins to change their specificity.

## Materials and methods

### Materials

Purified human coagulation proteases FXIa, thrombin, FXa, FXIIa, kallikrein, and APC were from Enzyme Research Labs (South Bend, IN, USA). Chromogenic substrates were purchased from Diapharma (West Chester, OH, USA): for FXIa and APC, S-2366; for kallikrein and FXIIa, S-2302; for thrombin, S-2238; and for FXa, S-2765.

### DNA manipulations

Standard methods of DNA restriction, ligation, electrophoresis, gel purification, ligation, and transformation of *E. coli* were as previously described^39,69^. Cloning steps used *E. coli* DH5α while *E. coli* TOP10 was used for protein expression and/or purification unless otherwise noted. All plasmids constructed in this study and used to express AAT variant proteins were verified by DNA sequencing by the Mobix Lab central facility, Faculty of Health Sciences, McMaster University, prior to use, throughout the AAT open reading frame. Oligodeoxyribonucleotides were synthesized by Integrated DNA Technologies (IDT, Coralville, IO, USA).

### Generation of phage display library hypervariable between AAT M358R P13 and P8

The AAT cDNA was first altered by Polymerase Chain Reaction (PCR) using template plasmid pUC19-API M358R^39^, oligonucleotide primers 5919AS and P13P8ranS (see Table S1 for the sequence of all oligonucleotides employed in this study) (note that any oligonucleotide named with the abbreviation “ran” (for randomized) contained all four deoxyribonucleotides at each position, in this case codons specifying P13 and P11-P8, with the P12 codon left unaltered) and Phusion High-Fidelity DNA polymerase under conditions recommended by the manufacturer (Thermo Fisher Scientific, Mississauga, ON, Canada). The PCR product was restricted with ClaI and HindIII and ligated between these sites in pUC19-API M358 to generate pUC19-API M358R-P13P8ran. The 1205 bp EcoRI-HindIII restriction fragment of pUC19-API M358R-P13P8ran was ligated to T7Select10-3b vector arms, packaged, and used to infect *E. coli* BLT5403 following the T7Select System manufacturer’s directions (Novagen, Madison, WI) as described^39^. The T7Select10-3b API M358R/P13P8ran library encoded phage-displayed AAT M358R with randomized P13 (E346X, where X is any amino acid), P12 fixed as A347, and P11-P8 randomized (AGAM A348X/G349X/A350X/M351X).

### Generation of plasmid library hypervariable at AAT M358R P2′/P3’

A bacterial expression library encoding AAT M358R randomized at P2′ and P3′ was constructed. First, PCR was performed using pBAD-H_6_α_1_PI M358R^69^ as the template and primers 5918S and P2′P3′ranAS and conditions described above. The PCR product was restricted with XagI and Bsu361 and inserted between the corresponding sites of pBAD-H_6_α_1_PI M358R to generate the pBAD-H_6_α_1_PI M358R P2′P3′ran. This library of plasmids directed arabinose-inducible cytoplasmic expression of AAT M358R/I356X/P355X proteins.

### Biopanning of phage display libraries

Both the previously reported T7Select10-3b API M358R(P7-P3ran)^39^ and T7Select10-3b API M358R/P13P8ran bacteriophage libraries were biopanned as previously described, except that purified FXIa was substituted for thrombin and biotinylated affinity-purified goat anti-human FXI IgG (Affinity Biologicals, Ancaster, ON, Canada) for anti-prothrombin antibodies cross-reacting with thrombin. Five rounds of biopanning with or without FXIa was performed in each case, starting with 10^9^ bacteriophages in liquid lysates. Phage reactive with FXIa (or mock-screened adherent phage) were captured using biotinylated antibodies and streptavidin-coated magnetic beads and the antibody-bead assembly was used to infect *E. coli* BLT5403 to start each subsequent round. After five rounds the AAT sequences of the hypervariable regions were characterized by either direct sequencing of plaque PCR samples or deep sequencing (see below).

### Bacterial lysate screening

Lysates of clonal colonies of *E. coli* TOP10 transformed with pBAD-AAT M358R P2′P3′ran were assayed for binding to purified proteases immobilized on microtiter plate wells, as previously described for thrombin^41^. For FXIa, the same procedure was employed, but the concentration was reduced to 0.1 µg/ml in the coating step.

### DNA sequencing

Variant RCL sequences of AAT variants identified via phage display or bacterial lysate screening were determined using dideoxy DNA sequencing of PCR products or plasmids as previously described^39,41^ for the P7-P3 and P2-P3′ screening experiments. For the P13-P8 screening effort, next generation (also known as deep) sequencing was employed. PCR was performed using phage lysates from: the unselected library; the Round 5 library selected with FXIa; and the Round 5 library mock selected without FXIa. Sense oligonucleotide P503 was used in each case, paired with antisense oligonucleotide P706, P708, and P709, respectively. Deep sequencing of these amplicons was performed at McMaster University’s Farncombe Metagenomics Facility, using an Illumina Nextera XT library kit; sequencing output was analyzed using the BioEdit sequence alignment editor and Microsoft Excel. The total number of reads was limited by sharing of the deep sequencing runs with appropriately barcoded samples from other investigators, as a cost-containment measure.

### Assembly of combined motif arabinose-inducible expression plasmids

AAT RCL sequence motifs with the highest activity and specificity from each screening exercise were combined as shown schematically in Fig. 1. In each case, synthetic DNA fragments (gBlocks; Integrated DNA Technologies, Kanata, ON, Canada) of 361 bp, containing within them two or three of the identified sequence motifs, were inserted between the BstXI and EcoRI sites of pBAD-H_6_α_1_PI M358R. Resultant plasmids encoded N-terminally hexahistidine tagged AAT M358R with substitutions were designated pBAD-AAT-R(A, B, C, or D) where “R” indicates AAT M358R and the letter in parentheses corresponds to the motif combination shown in Fig. 1 and Table 1.

### Assembly of GST-AAT M358R expression plasmids

Expression plasmid pGEX5X-1(GE Healthcare, Mississauga, ON, Canada) was first modified for cleavable AAT fusion. PCR using oligonucleotide primers GEX5pS and GEX5pAS and pGEX5X-1 as the template was carried out using conditions described above. The PCR product was restricted with NcoI and EcoNI and inserted between these sites in pGEX5X-1 to yield plasmid pGEX5P. This plasmid was restricted with NcoI and EcoRI, and its 4970 bp fragment was ligated to the 1211 bp NcoI-EcoRI digestion product of pBAD-H_6_α_1_PI M358R to form pGEX-AAT M358R. This plasmid directed the synthesis of a 630 amino acid GST-AAT M358R fusion protein in which GST C-terminal K218 was joined to Glu1 of AAT by peptide SDLEVLFQ-GPMGH_6_, with the PreScission Protease (GST-human rhinovirus (HRV) 3C protease fusion protein, GE Healthcare) recognition site underlined and its cleavage (between Q and G) represented by the dash. Similarly, expression vector background for other constructs was switched from pBAD to pGEX by analogous steps using these NcoI and EcoRI sites to generate pGEX-AAT M358R/X, where X is the motif of interest from each of the three screening exercises, or pGEX-AAT-RY, where Y is A, B, C, or D, with respect to multi-motif constructs described above for the arabinose-inducible system.

### Protein purification (arabinose-inducible system)

Hexahistidine-tagged AAT M358R was purified from sonicated clarified lysates of *E. coli* TOP10 cells transformed with pBAD-AAT M358R and derivative plasmids induced with arabinose as previously described, using nickel chelate affinity and ion exchange chromatography^41,69^.

### Protein purification (cleavable GST fusion system)

*Escherichia coli* BL21 (DE3) transformed with pGEX-AAT R(A,B,C, or D) or pGEX-AAT M358R/X were grown to OD_600nm_ of 0.7 and induced with 0.1 mM isopropyl β-D-1-thiogalactopyranoside (IPTG) for 3 h at 37 °C with shaking (225 rpm). Cells were harvested by centrifugation and resuspended in resuspension buffer (RB) (phosphate buffered saline (PBS) with protease inhibitors (Complete, Roche)), at 20 mL RB/l of induced cell culture pellet, then lysed by sonication as described. Triton X-100 was added to sonicated lysates (to 1%) and mixed for 15 min with rocking prior to centrifugation. Clarified supernatants (CS) were incubated with packed glutathione agarose beads equilibrated with RB (at a ratio of 10 mL CS/mL of beads) in batch, then transferred to columns and washed with 5 column volumes of RB. Beads were suspended in cleavage buffer (CB,50 mM Tris–HCl, pH 7.0, 150 mM sodium chloride, 1 mM ethylene diamine tetra-acetic acid) in batch and PreScission Protease was added (20 units/mL of beads). The protease/bead slurry was incubated, with end-over-end mixing, for 16–18 h at 4 °C, then transferred to a column and the flow-through was collected and diluted into loading buffer for nickel-chelate chromatography conducted exactly as described above for the arabinose-inducible system. In some experiments the glutathione agarose beads were stripped, after protease-mediated elution, with 50 mM Tris–HCl and 10 mM reduced glutathione, pH 8.0.

### Kinetic analysis

The second order rate constant of inhibition (k_2_) for different proteases (5–75 nM) was determined for all AAT variants at 37 °C by a discontinuous method, under pseudo-first order conditions comprising at least a 10:1 molar ratio of AAT variant to protease, essentially as described^69,70^. PPNE buffer [20 mM sodium phosphate pH 7.4, 100 mM NaCl, 0.1 mM EDTA, 0.1% polyethylene glycol 8000] was used in all cases. The natural logarithm of the ratio of initial protease activity divided by the residual protease activity after inhibition was plotted versus time, and the slope of the line of best fit obtained by linear regression (k_obs_) was divided by the initial AAT variant molar concentration to yield k_2_. The stoichiometry of inhibition (SI) for FXIa was determined as previously described for thrombin^41,69,70^. Briefly, varying ratios of AAT variants to FXIa were incubated at ambient temperature for 2 h, and residual FXIa activity was determined at 37 °C and plotted versus the ratio of AAT: FXIa. The line of best fit was regressed to zero residual FXIa activity to yield the SI.

### Diluted PT assay

The PT assay was modified by dilution of test reagents, similarly to the approach of de Maat et al*.*^37^. PT reagent STA Neoplastine CI Plus (Diagnostica Stago) was diluted 1:500 with 16.6 mM CaCl_2_. Normal human pooled plasma (NHPP, 50 µL) was as previously described and was supplemented with purified AAT variants and pre-warmed to 37 °C prior to starting the reaction by addition of 100 µL of diluted PT reagent. A STA-IV clotting analyzer was employed (Diagnostica Stago) for all clotting assays in this study.

### Diluted APTT assay

In this modified APTT assay, the STA PTTA reagent (Diagnostica Stago) was diluted 1:15 with Owren-Koller buffer (Diagnostica Stago). NHPP supplemented with AAT variant protein (50 µL) was combined with 50 µl of diluted reagent and pre-warmed to 37 °C. Clotting was initiated by addition of 50 µL of 25 mM CaCl_2_.

### FXI-dependent APTT assay

FXI-deficient human plasma (50 µL) (Affinity Biologicals, Ancaster, ON, Canada) was supplemented to 2 nM with purified FXIa and to 500 nM with AAT variant protein, then combined with 50 µL undiluted APTT reagent. Clotting was then initiated with CaCl_2_ as described above for the diluted APTT assay.

### Statistical analysis

Statistical software (InStat version 3.06 (GraphPad Software, San Diego CA)) was employed. Graphs were generated by using Prism 4.03 software (GraphPad Software). Multiple comparisons were performed using ANOVA with Tukey post-tests, while comparisons of only two data sets employed Welch-corrected t tests. Statistical significance was ascribed to results with a *p* value < 0.05. Unless otherwise stated, values reported herein are means ± SD.

### Images

Images of polyacrylamide gels or immunoblots were captured by scanning using a model XR GelDoc system (Bio-Rad Laboratories, Mississauga, ON, Canada). Images were labelled and saved in Tagged Image File (TIF) format using PhotoShop CS6 version 13 software (Adobe Systems Incorporated, San Jose, CA, USA).

## Supplementary Information

Supplementary Information 1.

## Data Availability

All datasets generated during and/or analysed during the current study are available from the corresponding author on reasonable request.
