# Supplementary material for: Identification of an alpha-1 antitrypsin variant with enhanced specificity for factor XIa by phage display, bacterial expression, and combinatorial mutagenesis
Source: Sci Rep. 2021 Mar 10;11:5565. doi: 10.1038/s41598-021-84618-7 (PMC7946950; doi:10.1038/s41598-021-84618-7)
Supplement: Supplementary file 1 — Supplementary Information 1. [file 41598_2021_84618_MOESM1_ESM.pdf]

Supplementary Information To:

Identification of an alpha-1 antitrypsin variant with enhanced specificity for factor XIa by phage display, bacterial expression, and combinatorial mutagenesis

Varsha Bhakta[1], Mostafa Hamada[2], Amy Nouanesengsy[2], Jessica Lapierre[2], Darian L. Perruzza[2], and William P. Sheffield[1,2*].

[1]Centre for Innovation, Canadian Blood Services, Hamilton, Ontario, Canada, and
[2]Department of Pathology and Molecular Medicine, McMaster University, Hamilton, Ontario, Canada.

Supplementary Tables

Table S1. Oligonucleotide primers employed in this study.

**Name***	**Sequence**	**Purpose**
5919AS	5'-GCTAAGCTTC ATTTTTGGGT GGGATTCACC AC-3'	P13-P8 library construction
P13P8ranS	5'-ACCATCGATG AGAAAGGGAC TNNNGCGNNN NNNNNNNNNT TTTTAGGCCA TACCC-3'	P13-P8 library construction
5918S	5'-GATCCGAATTC AGAGGATCCC CAGGGAGATG CT-3'	P2'/P3' library construction
P2'P3'ranAS	5'-CTTGACCTCA GGNNNNNNAG AGAACGTGGT ATGGCCTC-3'	P2'/P3' library construction
GEX5pS	5'-CGGGGATCCC ATGGGCCCCT GGAACAGAAC TTCCAGATCC ATTTTGGAGG-3'	Modification of pGEX5X-1 to introduce NcoI/BamHI sites and PreScission protease cleavage site
GEX5pS	5'-TCCCCTATAC TAGGTTATTG GAAAATTAAG GGC-3'	Modification of pGEX5X-1 to introduce NcoI/BamHI sites and PreScission protease cleavage site
P5-503S	5'-AATGATACGG CGACCACCGA GATCTACACT ATCCTCTACA CTCTTTCCCT ACACGACGCT CTTCCGATCT ACCATCGATG AGAAAGGG-3'	Generating amplicons for deep sequencing (all libraries)
P7-706AS	5'-CAAGCAGAAG ACGGCATACG AGATCATGCC TAGTGCTGGA GTTCAGACGT GTGCTCTTCC GATCTGTTGA ACTTGACCTC AGG-3'	Generating amplicons for P13-P8 phage display original library deep sequencing (all libraries)

Table S1, continued.

Name*	Sequence	Purpose
P7-708AS	5’- CAAGCA GAAGAC GGCATA CGAGAT GTAGAG AGGTGC TGGAGT TCAGAC GTGTGC TCTTCC GATCTG TTGAAC TTGACC TCAGG -3’	Generating amplicons for P13-P8 phage display original library deep sequencing (Round 5 + FXIa)
P7-709AS	5’-CAAGCAGAAG ACGGCATACG AGATCCTCTC TGGTGCTGGA GTTCAGACGT GTGCTCTTCC GATCTGTTGA ACTTGACCTC AGG -3’	Generating amplicons for P13-P8 phage display original library deep sequencing (Round 5 - FXIa)

*Oligonucleotides are designated “S” for sense and “AS” for anti-sense.

Table S2. Deep sequencing results from P13-P8 phage display screening.

Sample	P13-P8 translated sequence	Number of reads	Frequency (%)
Round 5 biopanned library (+ FXIa)	All	24362	100
	HASTGQ	13890	57.0
	IAKYAT	8111	33.3
	FASSSG	2073	8.5
	GAGVQF	103	0.4
	All others	206	0.8
Round 5 biopanned library (- FXIa)	All	31107	100
	FASSSG	30877	99.3
	FASSSE	14	0.5
	All others	216	0.2

Table S3. Alignment of AAT and serpin sequences

**Protein name/description**	**RCL Sequence (P14-P3')**	**Reported (in this study or Reference number in main manuscript)**
Antithrombin	S**EAA**ASTAVVIAG**RS**LN	50
Protease nexin 1	**T**K**A**SA**A**TTAIL**I**A**RS**S**P**	51
AAT M358R	**TEAAGAMFLEAIPRSIP**	30, 31
AAT M358R/HASTGQ	**T**H**A**STGQ**FLEAIPRSIP**	This study
AAT M358R/CLEVE	**TEAAGAM**C**LE**VE**PRSIP**	This study
AAT M358R/PRSTE	**TEAAGAMFLEAIPRS**TE	This study
AAT-RA	**T**H**A**STGQC**LE**VE**PRS**TE	This study
AAT-RB	**T**H**A**STGQC**LE**VE**PRSIP**	This study
AAT-RC	**TEAAGAM**C**LE**VE**PRS**TE	This study
AAT-RD	**T**H**A**STGQ**FLEAIPRS**TE	This study
Published AAT variant	**TEAAGAMFLE**IEG**RSIP**	52
Published AAT variant	**TEAAGAMFLE**SMT**RSIP**	37
Published AAT variant	R**EAAGAMFLEAIPRSIP**	53
Published AAT variant	**TEAAGAMFLEA**LN**RSIP**	45, 46
Published AAT variant	**TEAAGAMFLEA**TQ**RSIP**	58
Published AAT variant	**TEAAGAMFLEAI**K**R**K**IP**	35
Published AAT variant	**TEAAGAMFLE**SMTRV**IP**	37

Primary amino acid sequences of various AAT M358R derivatives between P14 (T345) and P3' (P361) are shown. Bold indicates AAT M358R sequence; regular indicates mutated residues.

Supplementary Figure Legends

Figure S1. Expression and purification of AAT M358R as a cleavable glutathione sulfotransferase (GST) fusion protein. E. coli BL21 bacterial cell cultures transformed with pGEX5P-AAT M358R were induced, pelleted, and resuspended, and aliquots corresponding to each stage of protein purification were electrophoresed on a reduced SDS-polyacrylamide gel and stained. Steps are descriptively labelled above the lanes, left to right, and numbered (below). Two columns were employed: glutathione agarose (ag.); and nickel-chelate ag., in that order. The overall input (sonicated suspension, lane 1) was centrifuged to yield the clarified lysate (lane 2) for loading onto glutathione ag, generating the flow-through (FT) (lane 3), and wash (lane 4). PreScission protease was used to elute the column by specific (overnight) proteolysis between the GST and AAT M358R domains (glutathione ag. protease elution, lane 5), for loading onto the nickel-chelate ag. column, generating the FT (lane 6) and wash (lane 7). The nickel-chelate ag. column was eluted with imidazole (lane 8) to generate the homogenous purified AAT M358R preparation. Lane 9 shows stripping of glutathione ag., after protease elution, with glutathione to show (top to bottom) residual uncleaved GST-AAT M358R, GST-PreScission protease (comigrates with AAT M358R) and GST. The figure is a composite of two gels electrophoresed and stained at the same time. Gaps between some lanes indicate that they were not contiguous on the original gels as loaded (unlike lanes 1-4 and 5-7 inclusive).

Figure S2. Reactions of AAT M358R P7-P3 motif 1 variants with FXIa and thrombin. A stained reduced 10% SDS-polyacrylamide gel is shown, with equivalent aliquots of reactions containing 1.0 µM AAT M358R or variants identified above the lanes, and 0.2 µM (+FIIa or +FXIa) or no

(No protease) protease. Reaction time was 2 minutes at 37°C. M, molecular weight markers are shown at right: 200; 150; 120; 100; 85; 70; 60; 50; 40; and 30 kDa.

Figure S3. Reactions of AAT P2-P3’motif 3 variants with FXIa and thrombin. Panel A shows a stained reduced 10% SDS-polyacrylamide gel, with equivalent aliquots of reactions containing 1.0 µM AAT M358R/PRSLD or AAT M358R/PRSTE variants identified below the lanes, and with 0.1 µM (+FIIa or +FXIa) or without (No protease) added protease. Reaction time was 1 minute at 37°C. M, molecular weight markers, are the same as in Figure S2. Panel B shows second order rate constants of inhibition (k2) of AAT M358R/PRSTE (black) or AAT M358R/PRSLD (grey) with FXIa. Bars show the mean of 7-8 determinations ± SD. Horizontal capped line between groups indicates ***$p < 0.001$ by Welch-corrected student’s t test.

Figure S4. Reactions of AAT M358R variants with multiple motif substitutions with FXIa and thrombin. E. coli transformed with pBAD plasmid constructs encoding the unfused AAT variants identified above the lanes were expressed following arabinose induction and recovered from clarified cell lysates, and AAT concentrations in the lysates were determined by ELISA and adjusted to 0.5 µM. Lysates were reacted with 0.1 µM FXIa and reacted for 2 minutes (Panel A) or 0.1 µM FIIa and reacted for 5 minutes (Panel B) prior to SDS-PAGE and transfer to immunoblots developed with anti-AAT antibodies; “+” lanes correspond to reactions with added protease and “-“ lanes to those without. Prestained molecular weight markers (PSM) correspond to 180; 130; 100; 70; 55; 40; 35; and 25 kDa. Panel B shows a single blot with contiguous lanes. All lanes in Panel A are from the same blot but gaps have been introduced to separate lanes that were not contiguous.

Supplementary Figures

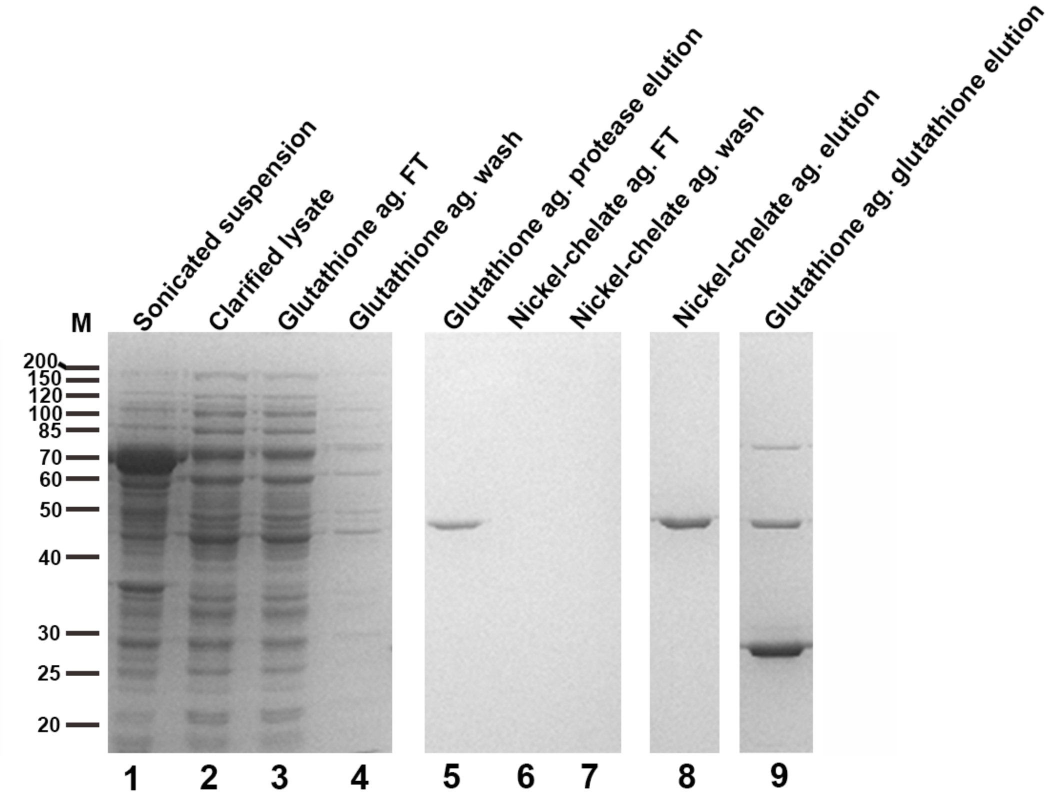

Figure S1.

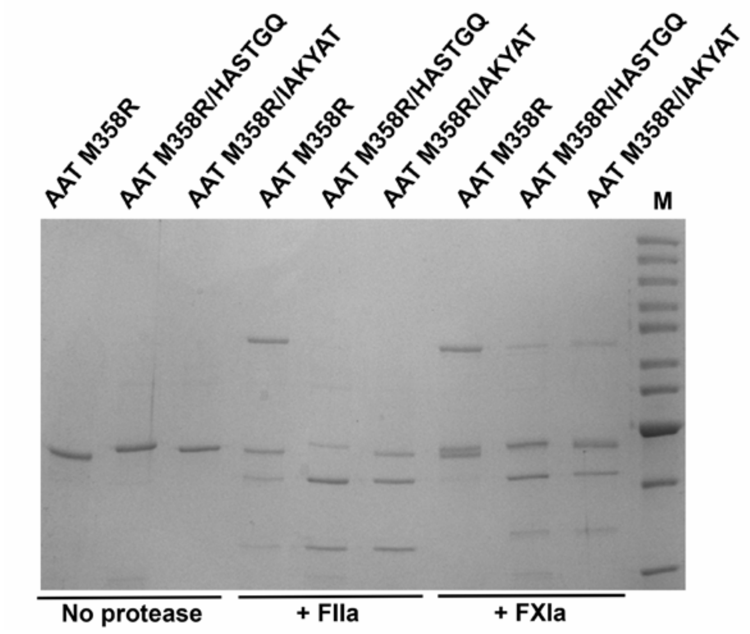

Figure S2.

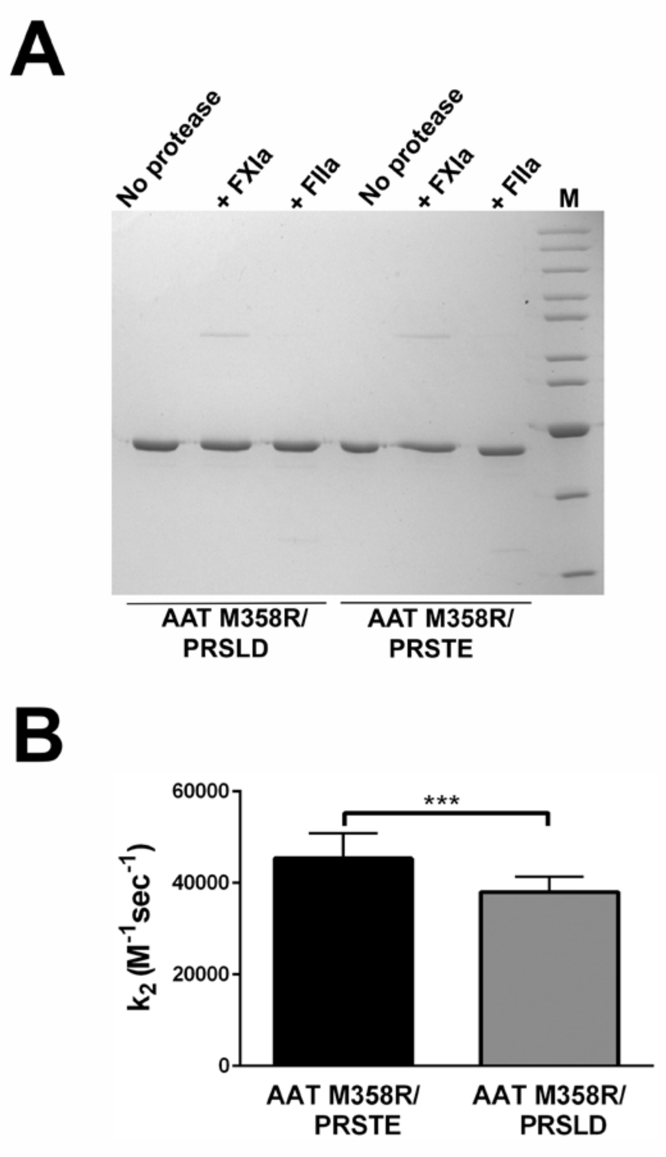
A
No protease
+ FXIa
+ FIIa
No protease
+ FXIa
+ FIIa
M
AAT M358R/
PRSLD
AAT M358R/
PRSTE
B
60000
40000
20000
0
k2 (M-1sec-1)
***
AAT M358R/
PRSTE
AAT M358R/
PRSLD

Figure S3.

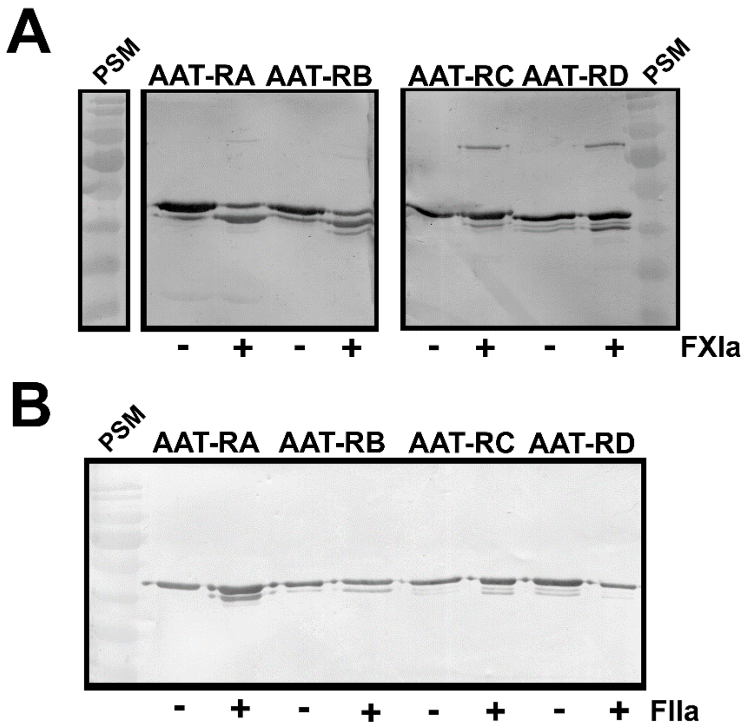
A
PSM
AAT-RA AAT-RB
AAT-RC AAT-RD
PSM
- + - + - + - + FXIa
B
PSM
AAT-RA AAT-RB AAT-RC AAT-RD
- + - + - + - + FIIa

Figure S4.
